# Reactive Oxygen Species Hydrogen Peroxide Mediates Kaposi's Sarcoma-Associated Herpesvirus Reactivation from Latency

**DOI:** 10.1371/journal.ppat.1002054

**Published:** 2011-05-19

**Authors:** Fengchun Ye, Fuchun Zhou, Roble G. Bedolla, Tiffany Jones, Xiufen Lei, Tao Kang, Moraima Guadalupe, Shou-Jiang Gao

**Affiliations:** 1 Tumor Virology Program, Greehey Children's Cancer Research Institute, The University of Texas Health Science Center at San Antonio, San Antonio, Texas, United States of America; 2 Department of Pediatrics, The University of Texas Health Science Center at San Antonio, San Antonio, Texas, United States of America; 3 Department of Molecular Medicine, The University of Texas Health Science Center at San Antonio, San Antonio, Texas, United States of America; 4 Cancer Therapy and Research Center, The University of Texas Health Science Center at San Antonio, San Antonio, Texas, United States of America; Oregon Health & Science University, United States of America

## Abstract

Kaposi's sarcoma-associated herpesvirus (KSHV) establishes a latent infection in the host following an acute infection. Reactivation from latency contributes to the development of KSHV-induced malignancies, which include Kaposi's sarcoma (KS), the most common cancer in untreated AIDS patients, primary effusion lymphoma and multicentric Castleman's disease. However, the physiological cues that trigger KSHV reactivation remain unclear. Here, we show that the reactive oxygen species (ROS) hydrogen peroxide (H_2_O_2_) induces KSHV reactivation from latency through both autocrine and paracrine signaling. Furthermore, KSHV spontaneous lytic replication, and KSHV reactivation from latency induced by oxidative stress, hypoxia, and proinflammatory and proangiogenic cytokines are mediated by H_2_O_2_. Mechanistically, H_2_O_2_ induction of KSHV reactivation depends on the activation of mitogen-activated protein kinase ERK1/2, JNK, and p38 pathways. Significantly, H_2_O_2_ scavengers N-acetyl-L-cysteine (NAC), catalase and glutathione inhibit KSHV lytic replication in culture. In a mouse model of KSHV-induced lymphoma, NAC effectively inhibits KSHV lytic replication and significantly prolongs the lifespan of the mice. These results directly relate KSHV reactivation to oxidative stress and inflammation, which are physiological hallmarks of KS patients. The discovery of this novel mechanism of KSHV reactivation indicates that antioxidants and anti-inflammation drugs could be promising preventive and therapeutic agents for effectively targeting KSHV replication and KSHV-related malignancies.

## Author Summary

Kaposi's sarcoma-associated herpesvirus (KSHV) is the etiologic agent of all clinical forms of Kaposi's sarcoma (KS) and several other malignancies. The life cycle of KSHV consists of latent and lytic phases. While establishment of viral latency is essential for KSHV to evade host immune surveillances, viral lytic replication promotes KSHV-induced malignancies. In this study, we show that the reactive oxygen species (ROS) hydrogen peroxide (H_2_O_2_) induces KSHV reactivation from latency. Furthermore, induction of KSHV reactivation by oxidative stress, hypoxia, and proinflammatory and proangiogenic cytokines, which are physiological hallmarks in all clinical forms of KS patients, is mediated by H_2_O_2_. Significantly, antioxidants inhibit H_2_O_2_-induced KSHV lytic replication in culture and in a mouse model of KSHV-induced lymphoma. These results show that ROS is likely an important physiological cue that triggers KSHV replication. The discovery of this novel mechanism of KSHV reactivation indicates that antioxidants and anti-inflammation drugs might be promising preventive and therapeutic agents for effectively targeting KSHV replication and KSHV-related malignancies.

## Introduction

A hallmark of herpesviral infections is the establishment of latency in the hosts following acute infections [1]. Reactivation of herpesviruses from latency results in production of infectious virions and often development of their associated diseases. KSHV is a gammaherpesvirus associated with KS, a vascular malignancy of endothelial cells commonly seen in AIDS patients [2]. KSHV is also linked to other lymphoproliferative diseases including primary effusion lymphoma (PEL) and multicentric Castleman's disease (MCD) [2]–[4]. Similar to other herpesviruses, KSHV establishes a lifelong persistent infection in the host [1]. In KS tumors, most tumor cells are latently infected by KSHV, indicating an essential role of viral latency in tumor development [5]. However, KSHV lytic replication also contributes to KS pathogenesis [6]. Both viral lytic products and *de novo* infection promote cell proliferation, invasion, angiogenesis, inflammation and vascular permeability [6]. In fact, higher KSHV lytic antibody titers and peripheral blood viral loads are correlated with high incidence and advanced stage of KS [7]–[13], and KS regressed following anti-herpesviral treatments that inhibit lytic replication [14], [15].

While several cellular pathways such as mitogen-activated protein kinase (MAPK) pathways and protein kinase C delta regulate KSHV lytic replication [16]–[20], the common physiological trigger that reactivates KSHV from latency in patients remains unclear. A number of factors including proinflammatory and proangiogenic cytokines [21], [22], hypoxia [23], HIV and its product Tat [24]–[26], coinfection with human cytomegalovirus and human herpesvirus 6 [27], [28], and the activation of toll-like receptors [29] can cause KSHV reactivation in cultures. However, none of them is likely the trigger in all the clinical scenarios, which include different forms of KS, PEL and MCD. The mechanisms by which these factors reactivate KSHV from latency also remain unclear.

There are four clinical forms of KS. Patients with all forms of KS are characterized by high levels of inflammation and oxidative stress [30], [31]. Classical KS, mostly seen in elderly men in the Mediterranean and Eastern European regions, is ubiquitously associated with high level of inflammation and oxidative stress because of its close link with aging [32]. In African endemic KS, excessive iron exposure due to high content of iron in the local soils coupled with bare foot walking is a possible cofactor that can induce inflammation and oxidative stress [33], [34]. In transplantation KS, inflammation and oxidative stress are common because of immunosuppression and organ rejection [35]. Patients with AIDS-related KS (AIDS-KS) have high levels of inflammation and oxidative stress as a result of host responses to HIV infection and chronic inflammation [36]. In all clinical forms of KS, inflammation and oxidative stress are also the hallmarks in the tumors [37]. Both PEL and MCD often coexist with KS, and are commonly seen in HIV-infected patients [6]. PEL is often found in elderly men, particularly in HIV-negative cases. Thus, similar to KS patients, these patients often have high levels of inflammation and oxidative stress. Since inflammation and oxidative stress induce ROS, and high level of ROS activates the MAPK pathways [38], we postulated that ROS, as a result of inflammation and oxidative stress, might mediate KSHV reactivation from latency.

The most common ROS molecule in non-immune cells is H_2_O_2_, which is mainly produced by mitochondria as a byproduct of oxidative metabolism [38]. Because high level of H_2_O_2_ is cytotoxic, cells express multiple antioxidant enzymes such as catalase and glutathione peroxidase to remove H_2_O_2_ so that it is below the detrimental threshold in normal condition. During oxidative stress, cells produce and release a large amount of H_2_O_2_ as a consequence of lost balance between its production and its scavenging [38]. During infections and inflammatory responses, host phagocytes such as macrophages and neutrophils produce and release excessive amounts of H_2_O_2_ [39]–[41]. Thus, KS patients are deemed to have high levels of H_2_O_2_. In this study, we investigated the physiological role of H_2_O_2_ on KSHV reactivation.

## Results

### H_2_O_2_ induces KSHV reactivation through both paracrine and autocrine mechanisms

**Figure 1:**
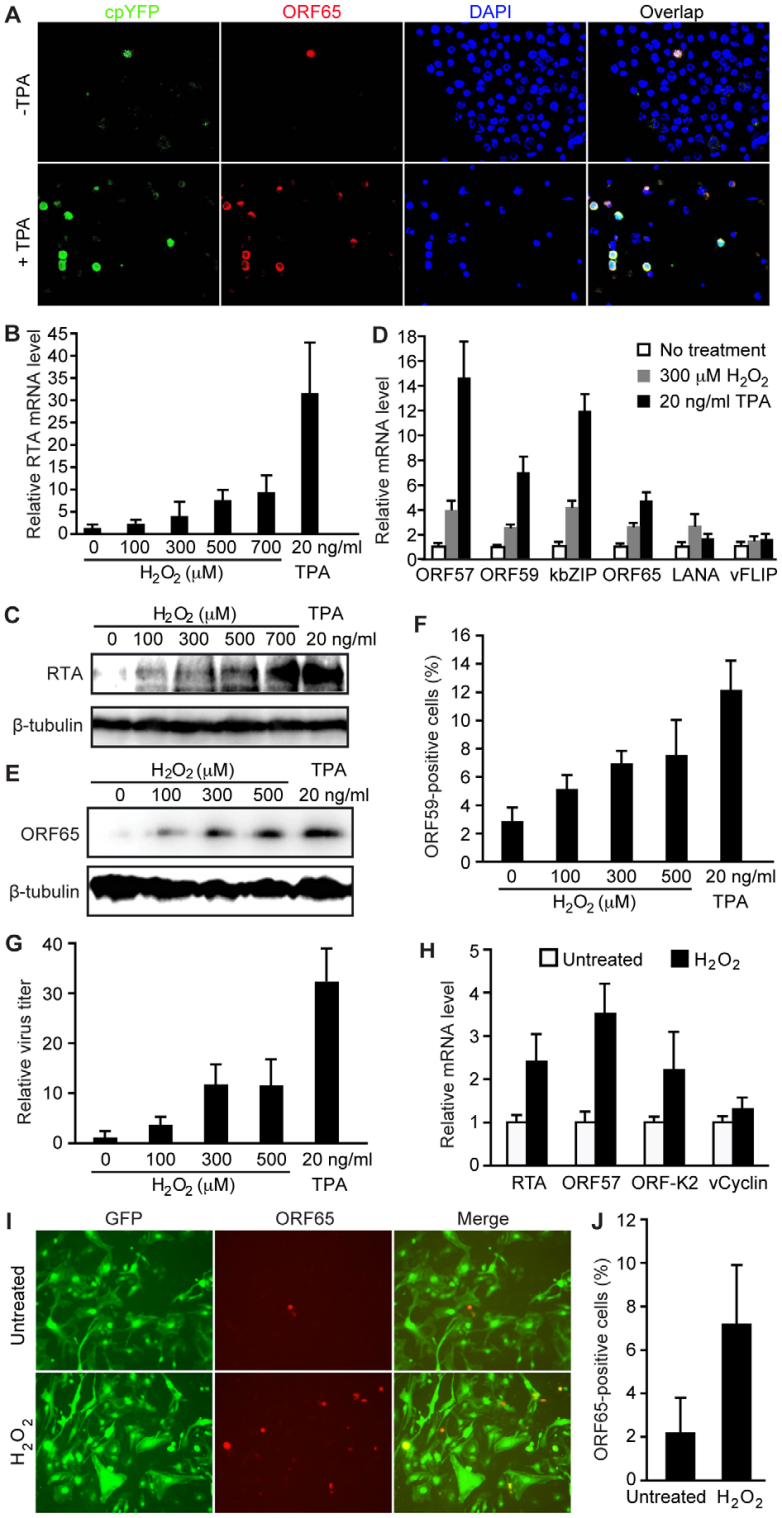
Exogenous H_2_O_2_ induces KSHV reactivation in PEL and endothelial cells. (A) Cells undergoing lytic replication in both uninduced BCBL1 cells and BCBL1 cells induced for lytic replication with phorbol ester TPA for 48 h also had high level of intracellular H_2_O_2_. Lytic cells were identified by staining for ORF65 protein (red), a late KSHV lytic protein while intracellular H_2_O_2_ level was monitored with a H_2_O_2_ sensor protein cpYFP. DAPI was used to label the nuclei. (B–C) Exogenous H_2_O_2_ induced the expression of KSHV RTA transcript and protein in BCBL1 cells in a dose-dependent manner. Cells were induced for 24 h. RTA transcript was detected by RT-qPCR (B). RTA protein was detected by Western-blotting usingβ-tubulin for calibration of sample loading (C). (D) Exogenous H_2_O_2_ induced the expression of KSHV lytic transcripts of ORF57, ORF59, kbZIP and ORF65 in BCBL1 cells detected by RT-qPCR while latent transcripts of LANA and vFLIP had minimal changes. Cells were induced for 24 h. (E–G) Exogenous H_2_O_2_ induced the expression of KSHV lytic proteins ORF65 and ORF59, and production of infectious virions in BCBL1 cells in a dose-dependent manner. ORF65 was detected by Western-blotting following 96 h of induction using β-tubulin for calibration of sample loading (E). ORF59 protein was detected by immunofluorescence staining following 48 h of induction (F). Relative virus titers were determined by using supernatants collected at 5 days of treatment to infect endothelial cells and calculating the numbers of GFP-positive cells at 48 hpi (G). (H) Exogenous H_2_O_2_ (150 µM) induced the expression of KSHV lytic transcripts of RTA, ORF57, ORF59, and ORF-K2 but not latent vCyclin transcript detected by RT-qPCR in latent KSHV-infected primary human umbilical vein endothelial cells (HUVEC). Cells were induced for 24 h. (I–J) Exogenous H_2_O_2_ (150 µM) induced the expression of KSHV lytic protein ORF65 in latent KSHV-infected HUVEC. ORF65 was detected by immunofluorescence staining following 96 h of induction (I). Quantification of ORF65-positive cells (J).

To examine the relationship of H_2_O_2_ with KSHV lytic replication, we stably expressed an H_2_O_2_-specific yellow fluorescent protein (cpYFP) sensor from the HyPer-cyto cassette in KSHV-infected BCBL1 cells [42]. As previously reported [20], the majority of BCBL1 cells were latently infected by KSHV but a small percentage of them underwent spontaneous lytic replication, which was detected by staining for viral late lytic protein ORF65 (Figure 1A). Notably, these ORF65-positive cells were strongly positive for cpYFP (Figure 1A). In contrast, ORF65-negative cells were either weakly positive or negative for cpYFP. Treatment with 12-*O*-tetradecanoylphorbol-13-acetate (TPA), a common chemical inducer for KSHV lytic replication, increased the number of lytic cells, all of which also expressed high level of cpYFP while ORF65-negative cells remained weakly positive for cpYFP (Figure 1A). Extended exposure of the images or adjustment of the contrast showed that almost all the TPA-treated cells were positive for cpYFP albeit with diverse intensities (data not shown). These diverse levels of H_2_O_2_ among the individual cells could be due to their different sizes of intracellular antioxidant enzyme pools. Together, these results showed a close correlation between high level of intracellular H_2_O_2_ and KSHV lytic replication.

To investigate the role of H_2_O_2_ in KSHV lytic replication, we examined whether exogenous H_2_O_2_, which uses water channels (aquaporins) to cross the cell membrane [43], is sufficient to induce KSHV reactivation. We observed a dose-dependent induction, at both mRNA and protein levels, of KSHV replication and transcription activator (RTA) encoded by ORF50, a key transactivator of viral lytic replication (Figure 1B–C), by H_2_O_2_. Consistent with these results, H_2_O_2_ increased the expression of several other KSHV lytic transcripts including ORF57, ORF59, kbZIP (ORF-K8) and ORF65 (Figure 1D). The expression of KSHV major latent gene LANA (ORF73) was also increased by 2.2-fold while that of another latent gene vFLIP (ORF71) remained almost unchanged. Furthermore, H_2_O_2_ increased the expression of viral lytic proteins ORF65 and ORF59, and production of infectious virions in a dose-dependent manner (Figure 1E–G).

We further extended the observation to primary human umbilical vein endothelial cells (HUVEC) latently infected by KSHV [44]. Similar to BCBL1 cells, H_2_O_2_ increased the expression of viral lytic transcripts including RTA, ORF57 and ORF-K2 but not latent transcript vCyclin (Figure 1H). H_2_O_2_ also increased the expression of ORF65 protein (Figure 1I–J). These results indicate that H_2_O_2_ induction of KSHV reactivation is not cell type specific.

**Figure 2:**
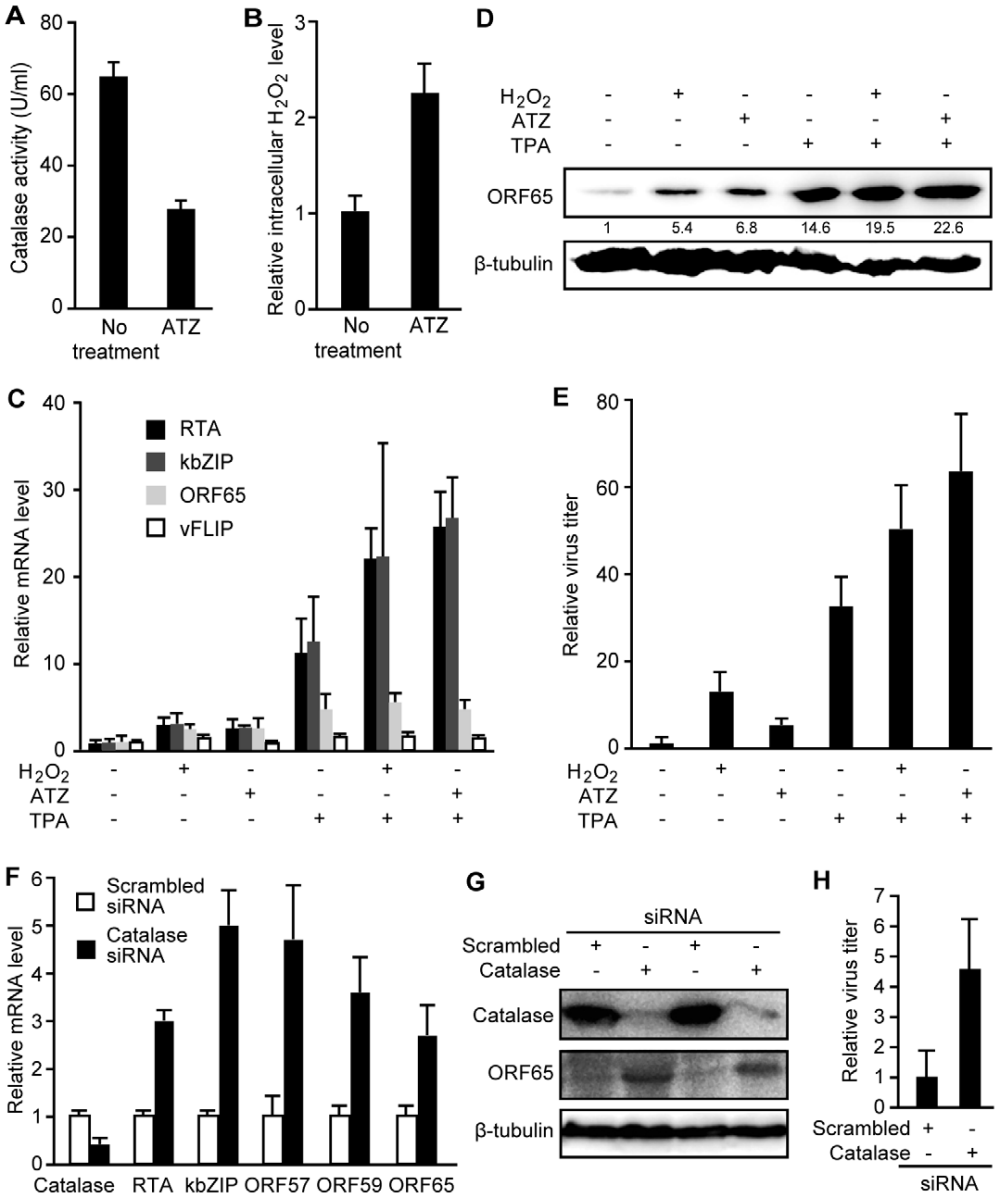
Intracellular ROS H_2_O_2_ induces KSHV reactivation. (A–B) Treatment of BCBL1 cells with catalase inhibitor ATZ reduced catalase activity (A) and increased intracellular H_2_O_2_ level (B). Cells were treated with 1 mM of ATZ for 12 h. (C–E) Induction of KSHV lytic replication in BCBL1 cells by treatment with ATZ, H_2_O_2_ and TPA alone or in combination. ATZ was used at 1 mM, H_2_O_2_ at 300 µM, and TPA at 20 ng/ml. KSHV RTA, kbZIP, ORF65 and vFLIP transcripts were detected by RT-qPCR following 24 h of treatment (C). Relative lytic protein ORF65 protein levels shown in numbers were detected by Western-blotting following 96 h of treatments using β-tubulin for calibration of sample loading (D). Relative virus titers were measured by using the supernatants to infect endothelial cells and calculating the numbers of GFP-positive cells at 48 hpi (E). (F–H) Silencing of catalase induced KSHV lytic replication. BCBL1 cells harboring BAC36 were stably transfected with siRNA specific to catalase or scrambled control. Transcripts of catalase, and KSHV RTA, kbZIP, ORF57, ORF59 and ORF65 were measured by RT-qPCR (F). Catalase, KSHV lytic protein ORF65, and β-tubulin were detected by Western-blotting (G). Relative virus titers were measured as described in “E” (H).

Next, we determined whether an increase in intracellular H_2_O_2_ level is sufficient to induce KSHV reactivation. Treatment of BCBL1 cells with 3-amino-1, 2, 4-triazole (ATZ), an inhibitor of H_2_O_2_ scavenging enzyme catalase, reduced cellular catalase activity by 57.2% and increased the intracellular H_2_O_2_ level by 2.2-fold (Figure 2A–B). ATZ increased the expression of KSHV lytic transcripts of RTA, ORF57, ORF59, kbZIP and ORF65 genes, the expression of lytic protein ORF65, and production of infectious virions (Figure 2C–E). Interestingly, we observed an additive effect when either H_2_O_2_ or ATZ was used together with TPA to induce KSHV lytic replication (Figure 2C–E).

To confirm that the effect of ATZ on KSHV reactivation was due to an increase in intracellular H_2_O_2_ level, we stably expressed a siRNA specific to catalase in BCBL1 cells harboring a recombinant KSHV BAC36 [45]. Compared to cells stably expressing a scrambled siRNA, those expressing the catalase-specific siRNA had significantly lower expression levels of catalase transcript and protein (Figure 2F–G). Similar to treatment with ATZ, knockdown of catalase increased the expression of KSHV lytic transcripts of RTA, ORF57, ORF59, kbZIP and ORF65, lytic protein ORF65, and production of infectious virions (Figure 2F–H).

Taken together, our results so far have shown that an increase in intracellular or exogenous H_2_O_2_ level induces KSHV reactivation, indicating that H_2_O_2_ produced during inflammation and oxidative stress in KS patients can be the physiological trigger that reactivates KSHV from latency through both autocrine and paracrine mechanisms.

### H_2_O_2_ scavengers inhibit H_2_O_2_-induced KSHV lytic replication

**Figure 3:**
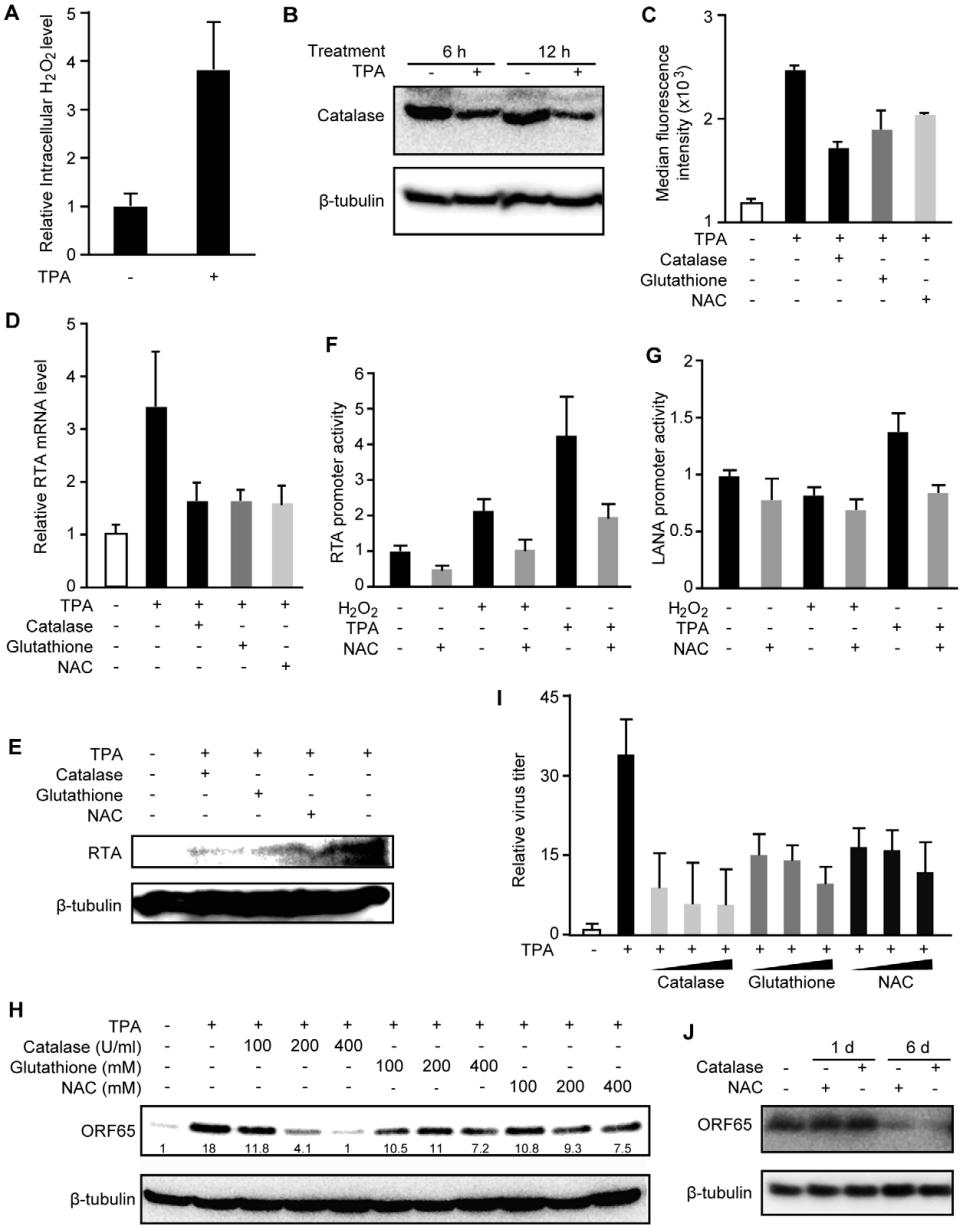
TPA-induced KSHV reactivation is mediated by H_2_O_2_. (A) Intracellular H_2_O_2_ levels in untreated BCBL1 cells and BCBL1 cells treated with TPA for 6 h. (B) Catalase protein levels in untreated BCBL1 cells and BCBL1 cells treated with TPA for 6 and 12 h. Catalase protein was detected by Western-blotting and calibrated with β-tubulin for sample loading. (C) H_2_O_2_ scavengers reduced TPA-induction of intracellular H_2_O_2_ levels measured by flow cytometry analysis of fluorescence intensity in BCBL1 cells stably expressing a H_2_O_2_ sensor protein cpYFP. Cells were treated with 20 ng/ml of TPA without any scavengers, or with scavenger catalase at 400 U/ml, reduced glutathione at 400 µM or NAC at 400 µM for 6 h. (D–E) H_2_O_2_ scavengers reduced TPA-induction of RTA transcript and protein in BCBL1 cells. Treatments of the cells were the same as in (C). RTA transcript was detected by RT-qPCR (D). RTA protein was detected by Western-blotting and calibrated with β-tubulin for sample loading (E). (F–G) H_2_O_2_ mediated the activation of lytic RTA but not latent LANA promoter. RTA promoter was induced by H_2_O_2_ and TPA, and this induction effect was inhibited by NAC (F). Latent LANA promoter was marginally activated by TPA but not by H_2_O_2_ (G). Luciferase activities were measured for 293 cells transfected with promoter reporter plasmids for 24 h, and treated with H_2_O_2_ (150 µM) or TPA (20 ng/ml) with or without NAC (400 µM) for 12 h. (I–H) H_2_O_2_ scavengers reduced TPA-induction of ORF65 protein and production of infectious virions in BCBL1 cells in a dose-dependent manner. TPA was used at 20 ng/ml. Relative ORF65 protein levels shown in numbers were detected following 72 h of treatment by Western-blotting and calibrated with β-tubulin for sample loading (H). Relative virus titers were determined by using supernatants collected at 5 days of treatment to infect endothelial cells and calculating the numbers of GFP-positive cells at 48 hpi (I). (J) H_2_O_2_ scavengers inhibited KSHV spontaneous lytic replication. ORF65 protein in BCBL1 cells treated with catalase (400 U/ml) and NAC (400 µM) for 1 and 6 day was determined by Western-blotting.

To determine whether H_2_O_2_ is required for KSHV lytic replication, we used H_2_O_2_ scavengers to reduce the intracellular H_2_O_2_ level. As shown in Figure 1A, TPA not only induced KSHV reactivation but also increased the intracellular H_2_O_2_ level. At 12 h, TPA increased the intracellular H_2_O_2_ level by 3.9-fold (Figure 3A), which could be the result of reduced expression of catalase (Figure 3B). Treatment with H_2_O_2_ scavengers including catalase, reduced glutathione and NAC inhibited TPA induction of intracellular H_2_O_2_ as shown by the reduced median fluorescent levels in the cpYFP-expressing BCBL1 cells (Figure 3C). None of these treatments affected the viability and growth rate of the cells (data not shown). As expected, H_2_O_2_ scavengers inhibited TPA induction of RTA transcript and protein (Figure 3D–E). Consistent with these results, RTA promoter activities were induced 2.2- and 4.3-fold by H_2_O_2_ and TPA, respectively, and these induction effects were inhibited by NAC (Figure 3F). In contrast, a latent LANA promoter was not induced by H_2_O_2_ and only marginally induced by TPA for 1.4-fold (Figure 3G). NAC also abolished TPA induction of the LANA promoter activity. Furthermore, TPA induction of ORF65 protein and production of infectious virions were inhibited by H_2_O_2_ scavengers in a dose-dependent fashion (Figure 3H–I). To examine whether H_2_O_2_ scavengers also inhibit KSHV spontaneous lytic replication, we measured the expression of ORF65 protein in BCBL1 cells treated with the scavengers. As shown in Figure 3J, both catalase and NAC inhibited the expression of ORF65 protein after 6 days but not 1 day of treatment, which is consistent with the late expression kinetics of this viral capsid protein. These results indicate that H_2_O_2_ is required for KSHV spontaneous lytic replication and TPA-induced KSHV reactivation, and antioxidants such as reduced glutathione and NAC can suppress KSHV lytic replication.

### H_2_O_2_ scavengers inhibit KSHV lytic replication induced by hypoxia, and proinflammatory and proangiogenic cytokines

**Figure 4:**
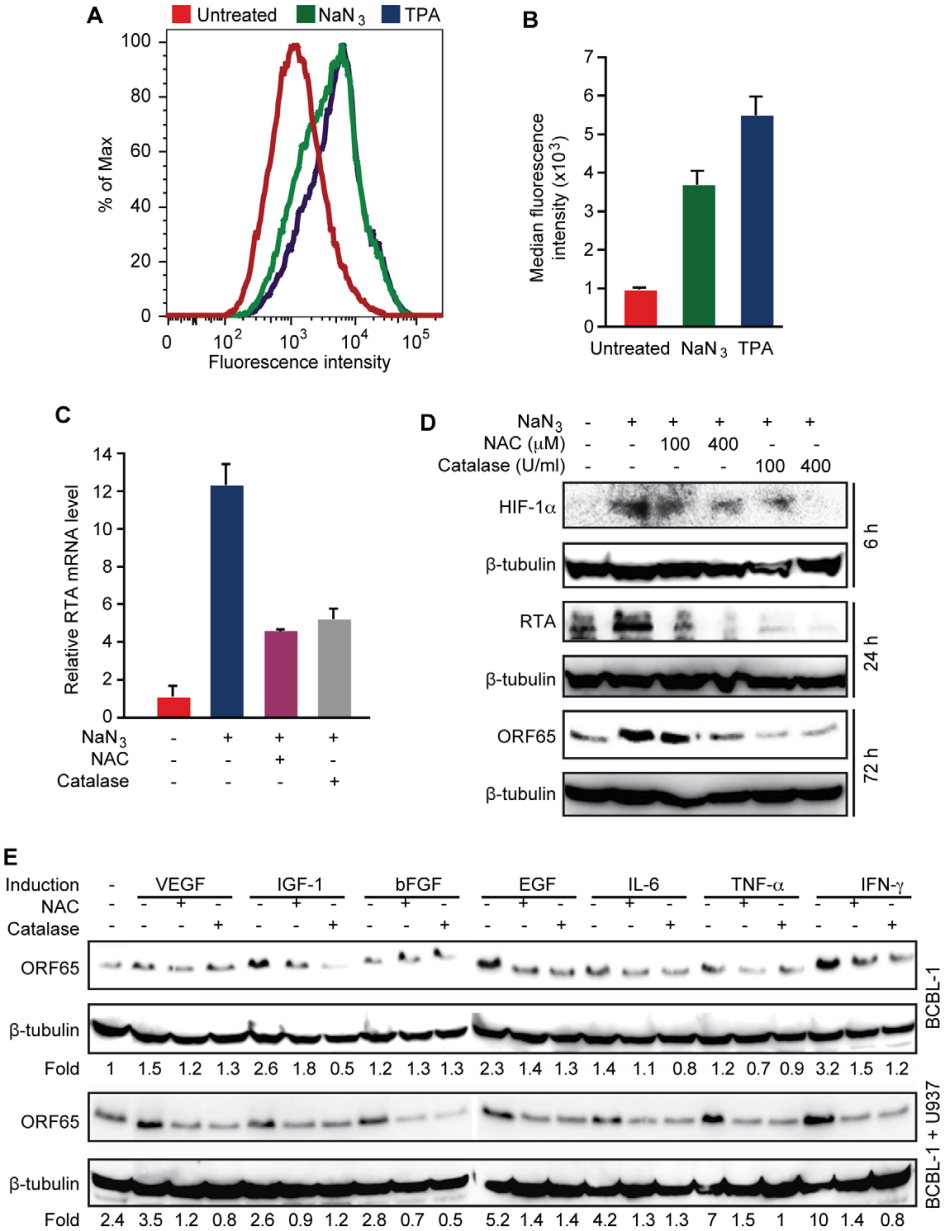
H_2_O_2_ mediates KSHV reactivation induced by hypoxia, and proinflammatory and proangiogenic cytokines. (A–B) Sodium azide (NaN**_3_**) and TPA increased intracellular H_2_O_2_ levels measured by flow cytometry analysis of fluorescence intensity in BCBL1 cells stably expressing a H_2_O_2_ sensor protein cpYFP. Cells were treated with NaN**_3_** (10 mM) for 90 min or 20 ng/ml of TPA for 12 h, and their fluorescence levels measured by flowcytometry shown in histogram (A) and median fluorescence intensity (B). (C) H_2_O_2_ scavengers inhibited NaN**_3_** induction of RTA transcript. BCBL1 cells were treated with NaN**_3_** (10 mM) for 90 min with and without NAC (400 µM) or catalase (400 U/ml). RTA transcript was detected by RT-qPCR following elimination of NaN**_3_**, and continuous culture with NAC or catalase for another 24 h. (D) H_2_O_2_ scavengers inhibited NaN**_3_** induction of HIF-1α, RTA and ORF65 proteins. BCBL1 cells were treated with NaN**_3_** (10 mM) for 90 min with and without NAC (400 µM) or catalase (400 U/ml). Following elimination of NaN**_3_**, cells were cultured with NAC or catalase, and Western-blotting was performed to measure the expression of HIF-1α protein at 6 h, RTA protein at 24 h, and ORF65 protein at 72 h. (E) H_2_O_2_ scavengers inhibited the expression of ORF65 protein in BCBL1 cells induced by proinflammatory and angiogenic cytokines. ORF65 protein was determined by Western-blotting in BCBL1 cells with or without co-culture with U973 cells treated with cytokines with or without NAC (400 µM) or catalase (400 U/ml) for 72 h. The concentrations of the cytokines were stated in the Materials and Methods. Relative ORF65 protein level was calculated using untreated BCBL1 cells as a reference (1-fold) and after calibration for protein loading with β-tubulin.

Because high levels of hypoxia, and proinflammatory and proangiogenic cytokines are features of KS tumors, and previous studies have shown that these conditions can induce KSHV reactivation [21]–[23], we determined whether KSHV reactivation induced by these conditions is mediated by H_2_O**_2_**. Short-time treatment with sodium azide (NaN**_3_**), which induces hypoxia [46], increased intracellular H_2_O_2_ level as shown by cpYFP fluorescent level in BCBL1 cells (Figure 4A–B). As a result, the expression of RTA transcript was increased 12.2-fold by NaN**_3_**, which was inhibited by both NAC and catalase (Figure 4C). Similarly, the expression of RTA and ORF65 proteins were induced by NaN**_3_**, which was also inhibited by NAC and catalase (Figure 4D). As expected, HIF-1α was induced by NaN**_3_**, which was also inhibited by NAC and catalase, suggesting that H_2_O_2_ mediates hypoxia induction of HIF-1α. These results are consistent with previous observations that H_2_O_2_ can directly induce HIF-1α [47].

Next, we determined the role of H_2_O_2_ in KSHV reactivation induced by proinflammatory and proangiogenic cytokines. Treatment of BCBL1 cells with vascular endothelial growth factor (VEGF), fibroblast growth factor-B (bFGF), interleukin-6 (IL-6) or tumor necrosis factor-alpha (TNF-α) alone minimally induced the expression of ORF65 protein by 1.5-, 1.2-, 1.4- and 1.2-fold, respectively, and these induction effects were reversed by NAC and catalase (Figure 4E). In contrast, insulin-like growth factor-1 (IGF-1), epithelial growth factor (EGF) and interferon gamma (IFN-γ) were more potent inducers, which increased the expression of ORF65 protein by 2.6-, 2.3- and 3.2-fold, respectively. Similarly, NAC and catalase inhibited the induction of ORF65 proteins by these cytokines. Since KS tumors contain abundant infiltration of proinflammatory immune cells such as monocytes, we further examined the effects of proinflammatory and proangiogenic cytokines on KSHV reactivation in the presence of monocytic cells U937 (Figure 4E). Co-culture of BCBL1 cells with U937 cells alone increased the expression of ORF65 protein by 2.4-fold. In the presence of U937 cells, weak inducers VEGF, bFGF, IL-6 and TNF-α more effectively increased the expression of ORF65 protein by 3.5-, 2.8-, 4.2- and 7-fold, respectively, suggesting a synergistic effect of these cytokines with U937 cells. These synergistic effects were also observed with strong inducers EGF and IFN-γ, which increased the expression of ORF65 protein by 5.2- and 10-fold in the presence of U937 cells. In contrast, IGF-1 did not further increase the expression of ORF65 protein in the presence of U937 cells. Both NAC and catalase inhibited the induction of ORF65 protein by proinflammatory and proangiogenic cytokines in the presence of U937 cells (Figure 4E). Together, these results indicate that H_2_O_2_ mediates KSHV reactivation induced by proinflammatory and proangiogenic cytokines with and without co-culture with the monocytic cells.

### H_2_O_2_ scavenger NAC inhibits KSHV lytic replication and tumor progression *in vivo*

**Figure 5:**
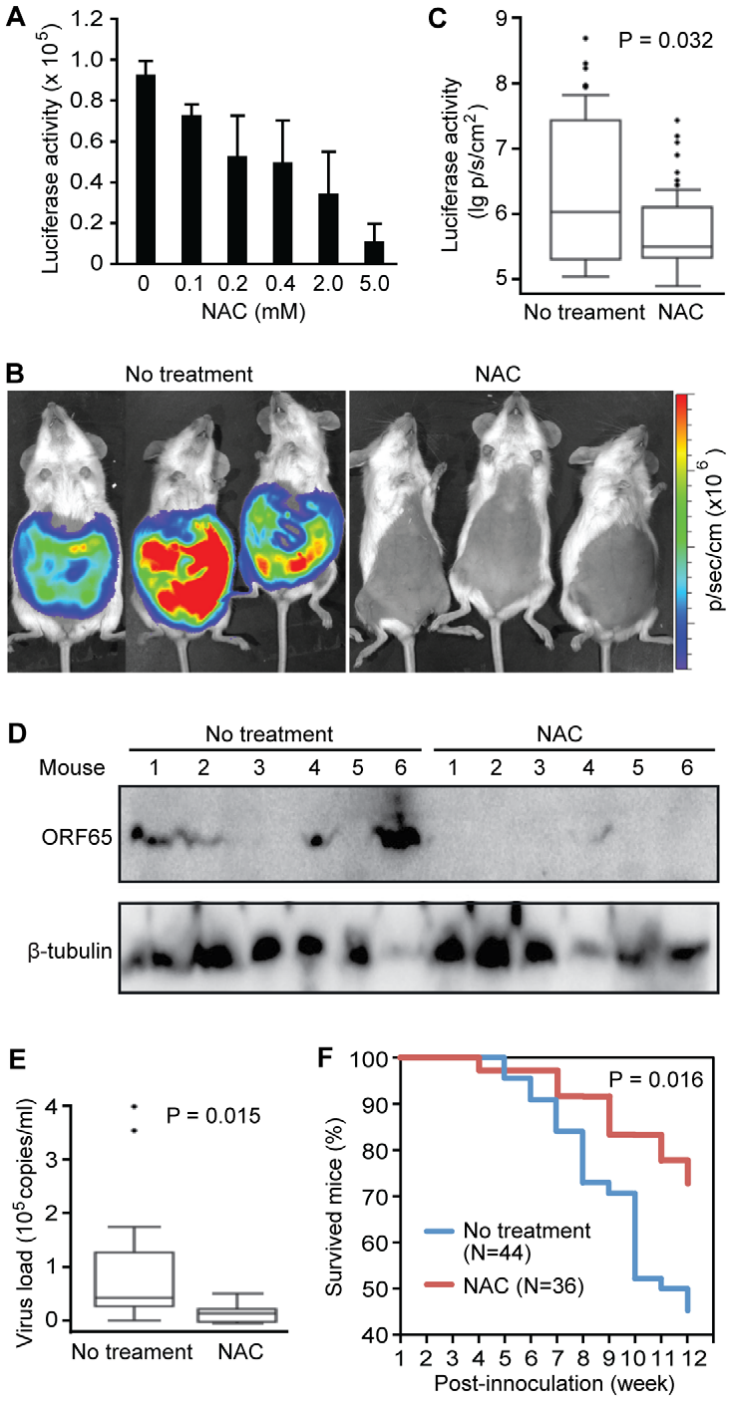
Antioxidant NAC inhibits spontaneous KSHV lytic replication *in vitro* and *in vivo*. (A) NAC inhibited the luciferase activities of BCBL1 cells harboring Δ65Luc in a dose-dependent fashion. The luciferase activities were measured following 72 h of treatment. (B-E) NAC inhibited KSHV lytic replication in a mouse PEL model. NOD/SCID mice intraperitoneally inoculated with BCBL1 cells harboring Δ65Luc at 5×10^6^ cells per mouse were examined for evidence of KSHV lytic replication. Representative images of the untreated control and NAC-treated groups of mice examined for luciferase activities five weeks after inoculation using a Xenogen IVIS 200 small animal imaging system (B). Luciferase activities of the untreated control (N = 44) and NAC-treated mice (N = 36) (C). Detection of KSHV ORF65 protein by Western-blotting in lymphoma cells from representative mice (D). Cells from 100 µl ascitic fluid from each mouse were examined. β-tubulin was used to calibrate the number of total cells. Relative viral loads in blood samples of control and NAC-treated mice (E). Kaplan-Meier analysis of the survival of the two groups of mice showing that NAC treatment significantly extended the lifespan of the mice compared to untreated mice (F).

We next sought to inhibit KSHV lytic replication *in vivo* with H_2_O_2_ scavengers. To monitor KSHV lytic activity *in vivo*, we generated a recombinant KSHV Δ65Luc by replacing ORF65 with a firefly luciferase gene (Figure S1A–C). Because ORF65 is a late viral lytic gene, detection of its expression would imply nearly complete KSHV lytic replication cycle. We reconstituted Δ65Luc in BCBL1 cells and generated a cell line harboring both wild type KSHV and Δ65Luc. As expected, BAC36-Δ65Luc cells expressed low level of ORF65 protein and luciferase, reflecting the spontaneous viral lytic replication in a small number of cells (Figure S1D). Treatment with TPA increased the expression of ORF65 and luciferase proteins. The corresponding luciferase activity was also increased by 6.2-fold (Figure S1E). These results indicate that the luciferase activity closely mimicked the expression of ORF65 protein, and thus can be used to monitor KSHV lytic activity. As expected, addition of NAC inhibited the luciferase activity in a dose-dependent manner (Figure 5A).

To examine KSHV lytic replication and determine the inhibitory effect of antioxidant NAC *in vivo*, we intraperitoneally inoculated NOD/SCID mice with BCBL1 cells harboring Δ65Luc. The mice were then fed daily with drinking water containing 5 mM NAC. All mice developed PEL at about five weeks post-inoculation as previously reported [48]. However, mice fed with NAC had an average 15.6-fold lower luciferase activities than those fed with drinking water alone (Figure 5B–C). We also detected lower expression levels of ORF65 protein in lymphoma cells isolated from the NAC-treated mice than those from the control mice by Western-blotting (Figure 5D). Immunohistochemical staining showed that the majority of the lymphoma cells from both groups were positive for LANA; however, cells from NAC-treated mice had significantly lower number of ORF65-positive cells than those from the control group (Figure S2A–B). To determine the production of infectious virions by the lymphoma cells, we used cell-free supernatants from the pleural fluids of the mice to infect HUVEC and examined the presence of infectious virions by staining for ORF65 protein [49]. We observed abundant virus particles in many of the cells infected with supernatants from the control mice while those infected with supernatants from NAC-treated mice had almost no detectable virus particles (Figure S2C). Consistent with these results, mice fed with NAC had an average 2.7-fold lower virus loads in the blood than the control group (Figure 5E). As many as 50% of the mice from both groups also developed solid tumors. ORF65 protein was detected in over 10% of the tumor cells from the control solid tumors but was almost not detectable in the solid tumors from the NAC-treated mice (Figure S2D). By examining the survival curves, we found that NAC-treated mice had an extended lifespan compared to the control group (Figure 5F). At 12-week post-inoculation, 72.2% of the NAC-treated mice survived compared to only 45.4% in the untreated group (P = 0.016). Collectively, results from these *in vivo* experiments indicated that PEL induced in mice had active KSHV lytic replication, and antioxidant NAC effectively inhibited KSHV lytic replication, and extended the lifespan of the mice.

### H_2_O_2_ induces KSHV reactivation by activating the ERK1/2, JNK and p38 MAPK pathways

**Figure 6:**
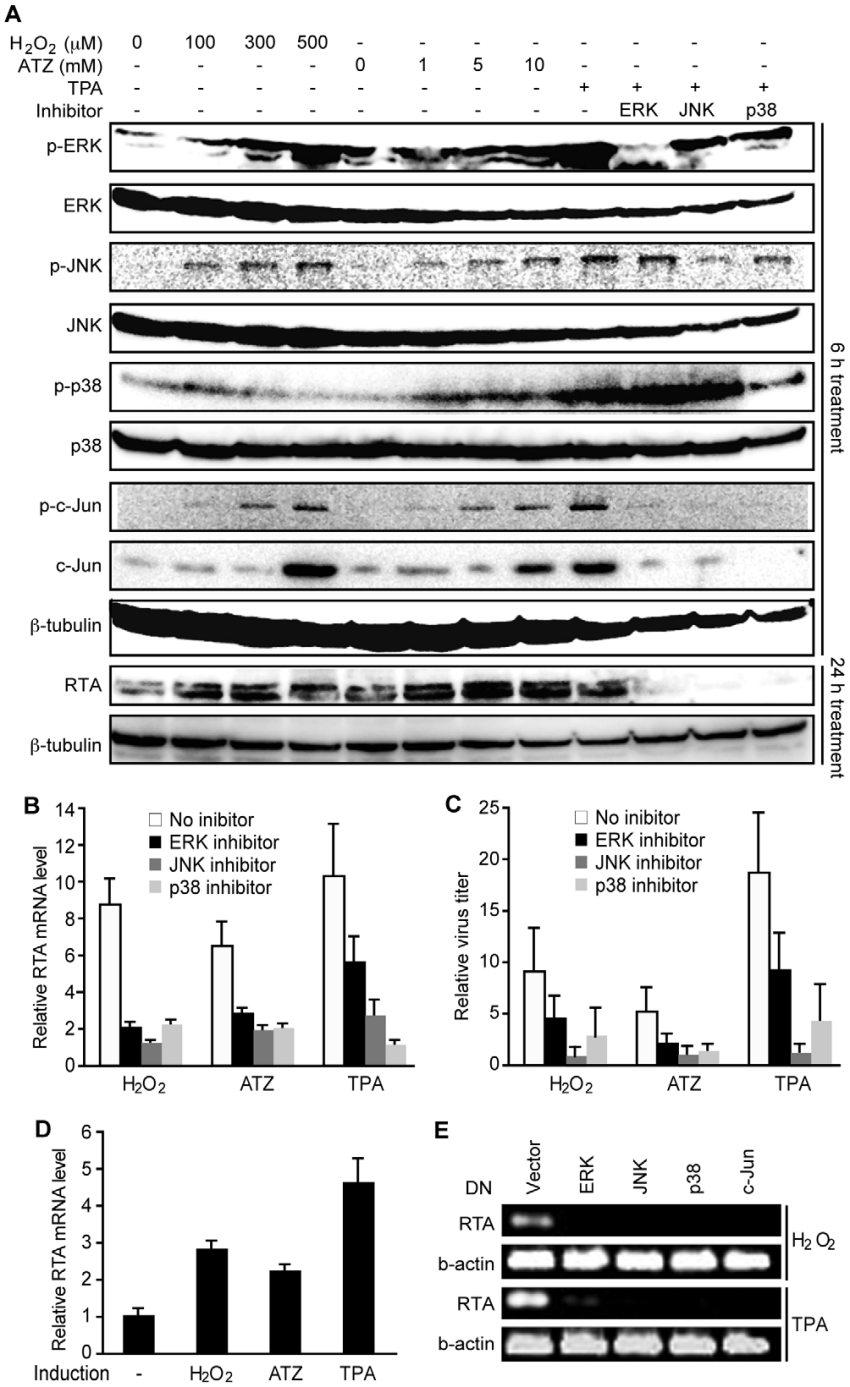
H_2_O_2_ induction of KSHV reactivation is mediated by ERK1/2, JNK, and p38 MAPK pathways. (A) Treatment with H_2_O_2_, ATZ or TPA activated ERK1/2, JNK and p38 pathways and their downstream transcriptional factor c-Jun, and induced the expression of RTA protein in BCBL1 cells harboring BAC36. Cells were treated with different concentrations of H_2_O_2_ and ATZ, or TPA at 20 ng/ml with DMSO control or inhibitors of MAPK pathways including 10 µM U0126 (ERK inhibitor), 50 µM JNK inhibitor II, and 50 µM SB203580 (p38 inhibitor) for 12 h. Total ERK1/2, JNK, p38 andc-Jun, and their phosphorylated forms, RTA protein, and β-tubulin were detected by Western-blotting. (B–C) Inhibitors of MAPK pathways inhibited the induction of RTA and production of infectious virions by H_2_O_2_, ATZ and TPA in BCBL1 cells harboring BAC36. H_2_O_2_, ATZ and TPA were used at concentrations of 300 µM, 5 mM and 20 ng/ml, respectively. Inhibitors of MAPK pathways were used at concentrations described in (A). Relative RTA transcript level at 24 h of treatment was detected by RT-qPCR with untreated cells set as “1” (B). Relative virus titers were determined by using supernatants collected at 5 days of treatment to infect endothelial cells and calculating the numbers of GFP-positive cells at 48 hpi (C). Virus titers from untreated cells were set as “1”. (D) H_2_O_2_ and ATZ, and TPA at concentrations of 300 µM, 5 mM and 20 ng/ml, respectively, induced the expression of RTA transcript in 293T cells harboring BAC36. RTA transcript was detected by RT-qPCR following 24 h of treatment. (E) Dominant negative (DN) constructs of MAPK pathways inhibited the induction of RTA transcript by H_2_O_2_ and TPA in 293T cells. 293T cells harboring BAC36 were transiently transfected with the control plasmid and DN constructs of ERK, JNK, p38, and c-Jun for 24 h, and treated with H_2_O_2_ at 300 µM and TPA at 20 ng/ml for an additional 12 h.

H_2_O_2_ is known to activate multiple MAPK pathways [50], which are required for KSHV lytic replication [17], [20]. Similar to TPA, both exogenous and ATZ-induced endogenous H_2_O_2_ activated ERK1/2, JNK, and p38 MAPK pathways, and increased the total and phosphorylated forms of their downstream target c-Jun in a dose-dependent manner in BCBL1 cells in addition to induction of RTA protein expression (Figure 6A). Treatment with specific inhibitors of all three MAPK pathways effectively inhibited TPA activation of their respective MAPKs and c-Jun, as well as the induction of RTA protein (Figure 6A). Importantly, these inhibitors also strongly inhibited H_2_O_2_ and ATZ induction of RTA expression and production of infectious virions (Figure 6B–C).

To further confirm the essential roles of MAPK pathways in H_2_O_2_-induced KSHV reactivation, we used dominant negative (DN) constructs to block these pathways. In 293T cells harboring BAC36, treatment with TPA induced KSHV reactivation [51]. Treatment with H_2_O_2_ and ATZ induced the expression of RTA transcript (Figure 6D). As expected, DN constructs of all three MAPK pathways and c-Jun effectively inhibited the induction of RTA by TPA and H_2_O_2_ (Figure 6E). Together, these results indicate that H_2_O_2_ induction of KSHV reactivation is mediated by all three MAPK pathways.

## Discussion

We have shown that ROS H_2_O_2_ induces KSHV lytic replication through both paracrine and autocrine mechanisms, and in both PEL and endothelial cells. Because oxidative stress and chronic inflammation are characteristic features in patients of all clinical forms of KS, as well as PEL and MCD [6], [30], [31], H_2_O_2_ could be an important physiological factor that triggers KSHV reactivation in these patients. Several other factors that induce KSHV lytic replication [21]–[29] also induce oxidative stress and inflammation [36], [52]–[56]. Thus, it is likely that H_2_O_2_ mediates KSHV lytic replication induced by these factors. Indeed, our data show that KSHV reactivation induced by oxidative stress, hypoxia, and proinflammatory and proangiogenic cytokines depends on the induction of H_2_O_2_. Importantly, co-culture of BCBL1 cells with monocytic U973 cells enhances KSHV reactivation induced by proinflammatory and proangiogenic cytokines, particularly IL-6, TNF-α and IFN-γ, which are highly expressed in KS tumors [6]. Because of the abundance of proinflammatory cells, and proinflammatory and proangiogenic cytokines in KS tumors, these synergistic effects could further boost KSHV lytic replication. Thus, tumor microenvironments consisting of proinflammatory cells, proinflammatory and proangiogenic cytokines, and possibly extracellular matrix and other stromal cells are likely to have essential roles in inducing and mediating KSHV lytic replication in KS tumors, which should be further examined in more details.

Mechanistically, we have shown that H_2_O_2_ induction of KSHV reactivation is mediated by ERK, JNK, and p38 MAPK pathways. Previous studies have shown that these pathways are required for KSHV infection and lytic replication [17]–[20]. Consistent with these observations, oxidative stress, hypoxia, and a number of proinflammatory and proangiogenic cytokines are known to induce MAPK pathways [57]–[65].

Based on our results, we propose a model in which KS tumors are initiated by either the homing of KSHV-infected cells, most likely progenitor endothelial cells or B-cells, from virus reservoirs to the affected sites or *de novo* infection by the newly produced virions, both of which are promoted by inflammation and oxidative stress [6]. Since KSHV *de novo* infection and lytic replication further promote inflammation and oxidative stress [6], one can expect the establishment of a positive feedback loop once the cycle is initiated. If these inflammatory conditions are not appropriately contained, they can lead to the rapid progression of KS as in the case of untreated AIDS-KS. Therefore, both active inflammation and host control of viral replication are likely to determine the course of KS.

It is interesting that only a subset of KSHV-infected cells undergo lytic replication in KS tumors or in cell culture induced for KSHV lytic replication (Figure 1A and F). KSHV has evolved a complex mechanism consisting of multiple blocks to regulate its replication [66]. Whether a cell undergoes lytic replication is likely to depend on the extent of release of these blocks. While KSHV lytic replication induces inflammation and promotes the overall tumor growth through a paracrine mechanism, it is also detrimental to the lytic cells [6]. Thus, a fine balance of latent and lytic programs in KS tumors combined with active inflammation and oxidative stress in the tumor microenvironment is likely required for the development of advanced stage of KS.

We have shown that H_2_O_2_ scavengers such as NAC can inhibit KSHV lytic replication *in vitro* and in a KSHV lymphoma animal model. Significantly, NAC extends the lifespan of the lymphoma-bearing mice. These results indicate that antioxidants and anti-inflammation drugs might be effective for inhibiting KSHV lytic replication, and thus could be promising preventive and therapeutic agents for KSHV-induced malignancies. Because many of these agents are affordable, their use is attractive, particularly in the African settings.

## Materials and Methods

### Cell culture and chemical reagents

BCBL1 cells and BCBL1 cells carrying pHyer-cyto or BAC36, a recombinant KSHV [45], were cultured in RPMI 1640 supplemented with 10% fetal bovine serum (FBS). Human embryonic kidney 293T cells, 293 cells and 293T cells carrying BAC36 were cultured in DMEM plus 10% FBS.

H_2_O_2_, ATZ, NaN**_3_**, and antioxidants NAC, reduced L-glutathione, and bovine liver catalase were purchased from Sigma Life Science (St. Louis, MO). The ERK inhibitor U0126, p38 inhibitor SB203580, and JNK inhibitor JNK inhibitor II were all purchased from Calbiochem (Gibbstown, NJ). TPA was from Sigma.

### Plasmids and lentiviruses

The p38 DN plasmid pcDNA3-p38/AF was provided by Jiahua Han at The Scripps Institute [67]. The JNK DN (HA-JNK1 [APF]) plasmid was from Lin Mantell at New York University School of Medicine [68]. The ERK DN (pCEP4L-HA-ERK1K71R) plasmid was from Melanie Cobb at the University of Texas Southwestern Medical Center [69]. The c-Jun DN plasmid pCMV-TAM67 was from Bradford W. Ozanne at Beatson Institute [70]. The pHyPer-cyto plasmid was purchased from Biocompare (South San Francisco, CA). Transfection of 293T and BCBL1 cells was carried out using the Lipofectamine LTX Reagent from Invitrogen (Carlsbad, CA). The lentiviruses expressing specific siRNA to human catalase and scrambled control were purchased from Santa Cruz (Santa Cruz, CA). BCBL1-BAC36 cells stably expressing catalase-specific or scrambled siRNAs were obtained following lentiviral infection and puromycin selection according to the instructions of the manufacturer.

### Measurement of cellular catalase activity and intracellular level of H_2_O_2_

A total of 5×10^6^ BCBL1 cells cultured with or without ATZ or TPA for 12 h were harvested by brief centrifugation. The cell pellets were homogenized in 0.5 ml ice cold phosphate saline buffer at pH 7.4 containing 1 mM EDTA. After centrifugation at 10,000 g for 15 min at 4°C, the supernatants were collected and used for measuring catalase activity or intracellular H_2_O_2_. Cellular catalase activity was determined with the OxiSelect Catalase Activity Assay Kit (Cell Biolabs, San Diego, CA), and intracellular H_2_O_2_ determined with the Fluorescent Hydrogen Peroxide/Peroxidase Detection Kit from Cell Technology (Columbia, MD). Alternatively, we tracked the intracellular H_2_O_2_ level with a H_2_O_2_ sensor by stable transfection of BCBL1 cells with a HyPer-cyto cassette consisting of a circularly permuted yellow fluorescent protein (cpYFP) under the control of the regulatory domain of the prokaryotic H_2_O_2_-sensing protein, OxyR [42].

### Induction of viral lytic replication and titration of infectious virions

To induce viral lytic replication, 2×10^6^ BCBL1 cells were treated with H_2_O_2_, ATZ or TPA alone, or in combination, in 10 ml RPMI 1640 medium containing 10% FBS for 48 h. The cells were then harvested, washed 1 time by centrifugation to eliminate the chemicals, and cultured in 5 ml fresh RPMI 1640 media with 10% FBS for three additional days. The supernatants were collected following centrifugation to eliminate cells and cell debris at 5,000 g for 15 min, and used for titration as previously described [44]. Relative virus titers were calculated based on the numbers of GFP-positive cells. Detection of virus particles in lymphoma supernatants from mice was carried out by staining for ORF65 with a monoclonal antibody following infection of HUVEC for 4 h as previously described [49].

To examine the effects of antioxidants on KSHV spontaneous lytic replication, BCBL1 cells were cultured in the presence NAC (400 µM) or catalase (400 U/ml) for 1 or 6 day, and cells were collected for Western-blotting analysis of ORF65 protein.

For induction of lytic replication by hypoxia, BCBL1 cells at 1.5×10^7^ cells/ml were treated with 10 mM NaN_3_ with or without antioxidants NAC (400 µM) and catalase (400 U/ml) for 90 min. Following washing by centrifugation, the cells were cultured for the specified lengths of time with or without NAC and catalase.

For induction of lytic replication by cytokines, BCBL1 cells at 1.5×10^7^ cells/ml were cultured in fresh medium with cytokines with or without antioxidants NAC (400 µM) and catalase (400 U/ml) for 72 h, and collected for Western-blotting. In parallel induction experiments, BCBL1 cells at 1.5×10^7^ cells/ml in fresh medium were induced with cytokines with or without antioxidants by co-culture with U937 cells at 5×10^4^ cells/ml pretreated with cytokines with or without antioxidants for 4 h. The following cytokines and concentrations were used: recombinant human VEGF at 200 ng/ml (Lonza, Walkersville, MD), recombinant human long R IGF-1 at 200 ng/ml (Lonza), recombinant human bFGF at 200 ng/ml (Lonza), recombinant human EGF at 200 ng/ml (Lonza), human IL-6 at 1 µg/ml (R&D Systems, Minneapolis, MN), human TNF-α at 1 µg/ml (R&D Systems) and human IFN-γ at 4000 U/ml (Sigma).

To induce KSHV lytic replication in endothelial cells, latent KSHV-infected HUVEC obtained as previously described [44] were treated with H_2_O_2_ (150 µM) for 24 h or 72 h, and cells were collected for RNA analysis or immunostaining for ORF65 protein, respectively.

### RNA extraction and quantitative reverse transcription real-time PCR (RT-qPCR)

RNA was purified using a Total RNA Purification Kit (Promega, Madison, WI). Total RNA (10 µg) was reversely transcribed into first-strand cDNAs by using a Superscript III First-Strand cDNA Synthesis Kit (Invitrogen). RT-qPCR was carried out in a DNA Engine Opticon 2 Continuous Fluorescence Detector (Bio-Rad, Hercules, CA). Each sample was measured in triplicate. The expression level of each transcript (mRNA) was first normalized to β-actin mRNA as previously described [71]. The relative expression level of a transcript in the treated cells was compared to the untreated cells, and calculated as fold changes. Specific primers for all KSHV genes were previously described [71]. Primers for human catalase were: 5′aggactaccctctcatcccagttg3′ (forward) and 5′gggtcccaggcgatggcggtgag3′ (reverse).

### Immunofluorescence antibody assay (IFA), immunohistochemistry and Western-blotting

KSHV lytic proteins ORF59 or ORF65 in BCBL1 cells were detected by IFA as previously described [51]. Expression of KSHV lytic proteins in lymphomas and solid tumors in mice were detected by immunohistochemistry. Briefly, cells from PEL induced in NOD/SCID mice were collected by centrifugation at 1,000 g for 5 min, fixed with formalin, and embedded in paraffin. Sections at 5 nm cut from the paraffin blocks were deparaffinized at 60°C, cleared, and rehydrated in xylene and graded alcohols. Antigen retrieval was done with citrate buffer at pH 6 for 20 min at 121°C in a pressure chamber. Sections were blocked successively with 3% H_2_O_2_ and bovine serum albumin buffer. Sections were then incubated with a monoclonal antibody to ORF65 or a mouse immunoglobulin fraction (DAKO, Carpinteria, CA) as a negative control for 1 h at 25°C. After three washes with PBS, the slides were further incubated with a secondary antibody conjugated to horseradish peroxidase (DAKO) for 15 min. The slides were then incubated with the diaminobenzidine substrate (DAKO), counterstained with hematoxylin, and mounted for observation.

Western-blotting was carried out as previously described [51]. The rabbit polyclonal antibodies to ERK1, p-ERK (Tyr 204), JNK1, p-JNK (Thr 183/Tyr 185), p38, p-p38 (Tyr 182), c-Jun, and catalase were from Santa Cruz; a polyclonal antibody to p-c-Jun (Ser73) was from Calbiochem; and a monoclonal antibody to β-tubulin was from Sigma. A rabbit polyclonal antibody to KSHV lytic protein RTA was a generous gift from Dr. Charles Wood at the University of Nebraska, Lincoln.

### Reporter assay

293 cells transfected with either the RTA promoter luciferase reporter plasmid or the latent LANA promoter (LTd) luciferase reporter plasmid using Lipofectamine-2000 Transfection Reagent (Invitrogen) for 24 h were treated with H_2_O_2_ (300 µM) or TPA (20 ng/ml) with or without antioxidants NAC (400 µM) or catalase (400 U/ml) for 12 h. Cells were collected and their luciferase activities determined as previously described [51]. Transfection efficiency was calibrated by co-transfection with the pSV-β-galactosidase construct (Promega).

### Detection of blood viral loads in mice

Cell-free DNA was isolated from two drops of blood from each mouse collected by tail bleeding using the QiAamp DNA Blood Mini Kit (Qiagen). KSHV DNA was detected by real-time PCR using vCyclin (ORF72) primers and purified BAC36 as copy number control as previously described [71]. KSHV viral loads expressed as genome copies per ml of blood were calculated. PCR assay for human β-actin gene was also carried out for these DNA samples to monitor the absence of any contamination of human cells [71]. None of the samples had any detectable signal for human β-actin gene.

### Generation of a recombinant KSHV expressing firefly luciferase under the control of the late lytic ORF65 promoter

A recombinant KSHV genome with the entire ORF65-coding frame deleted and replaced with the firefly luciferase gene was constructed using a “two-step” homologous recombination strategy as previously described (Figure S1A) [51]. Firstly, the firefly luciferase gene was amplified from the luciferase reporter plasmid pGL3-Basic Luciferase Reporter Vector (Promega) using primers 5′ttctcgagatggaagacgccaaaaacataaagaaaggcccg3′ (luciferase forward) and 5′ctcgagttaattaattacacggcgatctttccgcccttc3′ (luciferase reverse). The Kanamycin resistance cassette (Kan^R^) flanked by two LoxP sites was amplified from the transposon EZ-Tn5™ <Kan-2> (Epicenter, Madison, WI) using primers 5′tttttaattaagtgtaggctggagctgcttc3′ (Kan^R^ forward) and 5′ttttttaattaacatatgaatatcctccttag3′ (Kan^R^ reverse). The two PCR products were ligated using a T4 DNA ligase (New England Biolabs, Ipswich, MA). The resulting fragment was then used as a template to generate the Kan^R^-Luc cassette by PCR amplification using primers 5′cttgtgactccacggttgtccaatcgttgcctatttctttttgccagagg tttttaattaagtgtaggctggagctgcttc3′ (forward) and 5′aggtgagagaccccgtgatccaggagcgactggatcatgactacgctcac ttctcgagatggaagacgccaaaaacataaagaaaggcccg3′ (reverse). This PCR product, flanked by a 50 bp sequence from the immediate downstream region of ORF65 at its 5′end and a 50 bp sequence from the start codon (ATG) region of ORF65 at its 3′end, was electroporated into *Escherichia coli* strain DH10B containing recombinant KSHV BAC36 [45]. Upon homologous recombination, the Kan^R^-Luc cassette was integrated into KSHV genome. The Kanamycin-resistant colonies, containing the mutant KSHV genome, named Δ65Kan-Luc, with ORF65 replaced with Kan^R^-Luc cassette, were selected. To eliminate the Kan^R^ cassette, a Cre-expression plasmid pCre carrying a tetracycline resistant marker and a temperature (37°C)-sensitive replication origin was electroporated into the selected bacteria. The expression of Cre protein led to the removal of Kan^R^ cassette by LoxP-mediated recombination. The resulting colonies containing the mutant KSHV genome, named Δ65Luc, with ORF65 replaced with firefly luciferase gene, which was Tetracycline resistant but Kanamycin sensitive, were selected. The pCre plasmid was removed by culturing the bacteria at 37°C.

The mutant KSHV genomes were purified using the Large Construct DNA Purification Kit (Qiagen, Valencia, CA), verified for integrity by restriction digestion and PCR amplification of specific genes (Figure S1B–C), and electroporated into BCBL1 cells as previously described [45]. Following Hygromycin selection, a cell line harboring both the wild type KSHV genome and Δ65Luc was established. Expression of luciferase by this cell line was confirmed by Western-blotting using a luciferase-specific antibody (Figure S1D), and by measuring luciferase activity using the firefly luciferase substrate (Promega) and a Veritas Microplate Luminometer (Turner BioSystems, Sunnyvale, CA) (Figure S1E).

### Examination of the effect of antioxidant NAC on KSHV lytic replication in a mouse lymphoma model

This study was carried out in strict accordance with the recommendations in the Guide for the Care and Use of Laboratory Animals of the National Institutes of Health. The protocol was approved by the Institutional Animal Care and Use Committee (IACUC) at the University of Texas Health Science Center at San Antonio (Animal Welfare Assurance Number: A3345-01). All surgery was performed under sodium pentobarbital anesthesia, and all efforts were made to minimize suffering.

Male NOD/SCID mice at 6 weeks from Jackson Laboratories (Bar Harbor, ME) were intraperitoneally inoculated with BCBL1 cells carrying Δ65Luc at 5×10^6^ per mouse. One week after inoculation, mice (n = 36) were given drinking water supplemented with 5 mM of NAC while the control mice (n = 44) were given drinking water without the antioxidant. Mice were monitored daily for “PEL-like” symptoms. Luciferase activity and GFP intensity were measured five weeks after injection using a Xenogen IVIS 200 Imaging System (Xenogen, Alameda, CA). Mice were monitored daily, and terminated when they became immobile. Lymphoma cells, supernatants and solid tumors were collected and analyzed as indicated.

## Supporting Information

Figure S1 Construction of a recombinant KSHV with ORF65 replaced with firefly luciferase gene. (A) Schematic illustration of the “two-steps” recombination strategy for generating the mutant KSHV genome. (B) Genetic analysis of wild type BAC36, intermediate mutant Δ65Kan-Luc and recombinant virus Δ65Luc genomes by restriction digestion with Hind III. (C) Confirmation of the replacement of ORF65 by luciferase gene in intermediate mutant Δ65Kan-Luc and recombinant virus Δ65Luc genomes by PCR amplification. (D) Detection of luciferase and ORF65 proteins in uninduced and TPA-induced BCBL1 cells harboring Δ65Luc by Western-blotting. β-tubulin was used for the calibration of sample loading. TPA treatment was carried out for 72 h. (E) Detection of luciferase activities in uninduced and TPA-induced BCBL1 cells harboring Δ65Luc. TPA treatment was carried out for 72 h.
(TIF)

Figure S2 NAC treatment inhibits KSHV lytic replication in a mouse PEL model. (A) Representative immunohistochemistry images of ORF65 and LANA staining in lymphoma cells from untreated control and NAC-treated mice. (B) Percentages of ORF65-positive cells in lymphomas from untreated control and NAC-treated mice. (C) Detection of KSHV particles by ORF65 staining in endothelial cells infected with supernatants of lymphomas from untreated control and NAC-treated mice. Immunofluorescence staining was performed at 4 hpi. (D) Representative immunohistochemistry images of ORF65 staining in solid tumors from untreated control and NAC-treated mice.
(TIF)
