# Supplementary figures and images for: Reactive Oxygen Species Hydrogen Peroxide Mediates Kaposi's Sarcoma-Associated Herpesvirus Reactivation from Latency
Source: PLoS Pathog. 2011 May 19;7(5):e1002054. doi: 10.1371/journal.ppat.1002054 (PMC3098240; doi:10.1371/journal.ppat.1002054)

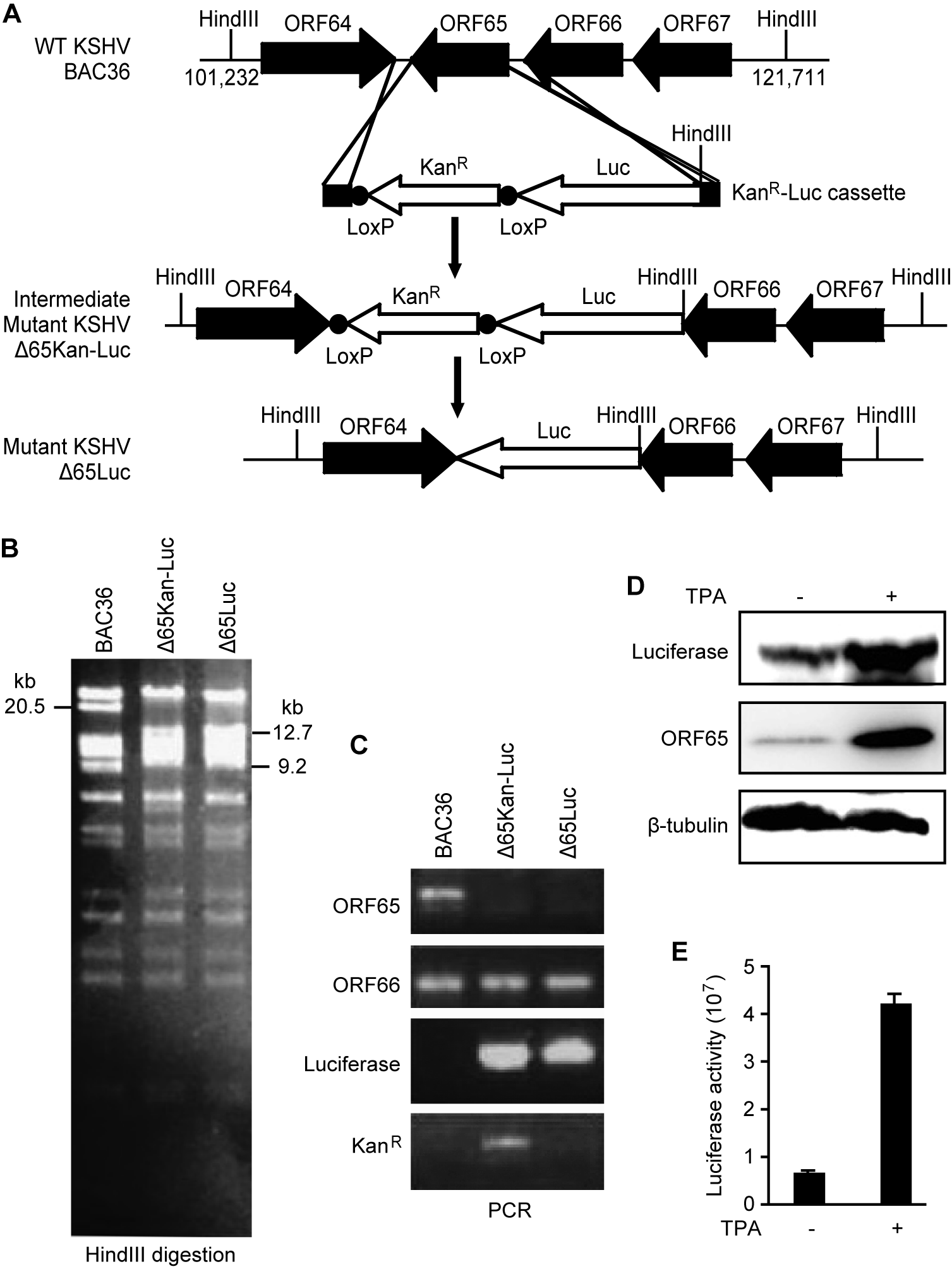

Supplement: Figure S1 — Construction of a recombinant KSHV with ORF65 replaced with firefly luciferase gene. (A) Schematic illustration of the “two-steps” recombination strategy for generating the mutant KSHV genome. (B) Genetic analysis of wild type BAC36, intermediate mutant Δ65Kan-Luc and recombinant virus Δ65Luc genomes by restriction digestion with Hind III. (C) Confirmation of the replacement of ORF65 by luciferase gene in intermediate mutant Δ65Kan-Luc and recombinant virus Δ65Luc genomes by PCR amplification. (D) Detection of luciferase and ORF65 proteins in uninduced and TPA-induced BCBL1 cells harboring Δ65Luc by Western-blotting. β-tubulin was used for the calibration of sample loading. TPA treatment was carried out for 72 h. (E) Detection of luciferase activities in uninduced and TPA-induced BCBL1 cells harboring Δ65Luc. TPA treatment was carried out for 72 h. (TIF) [file ppat.1002054.s001.tif]

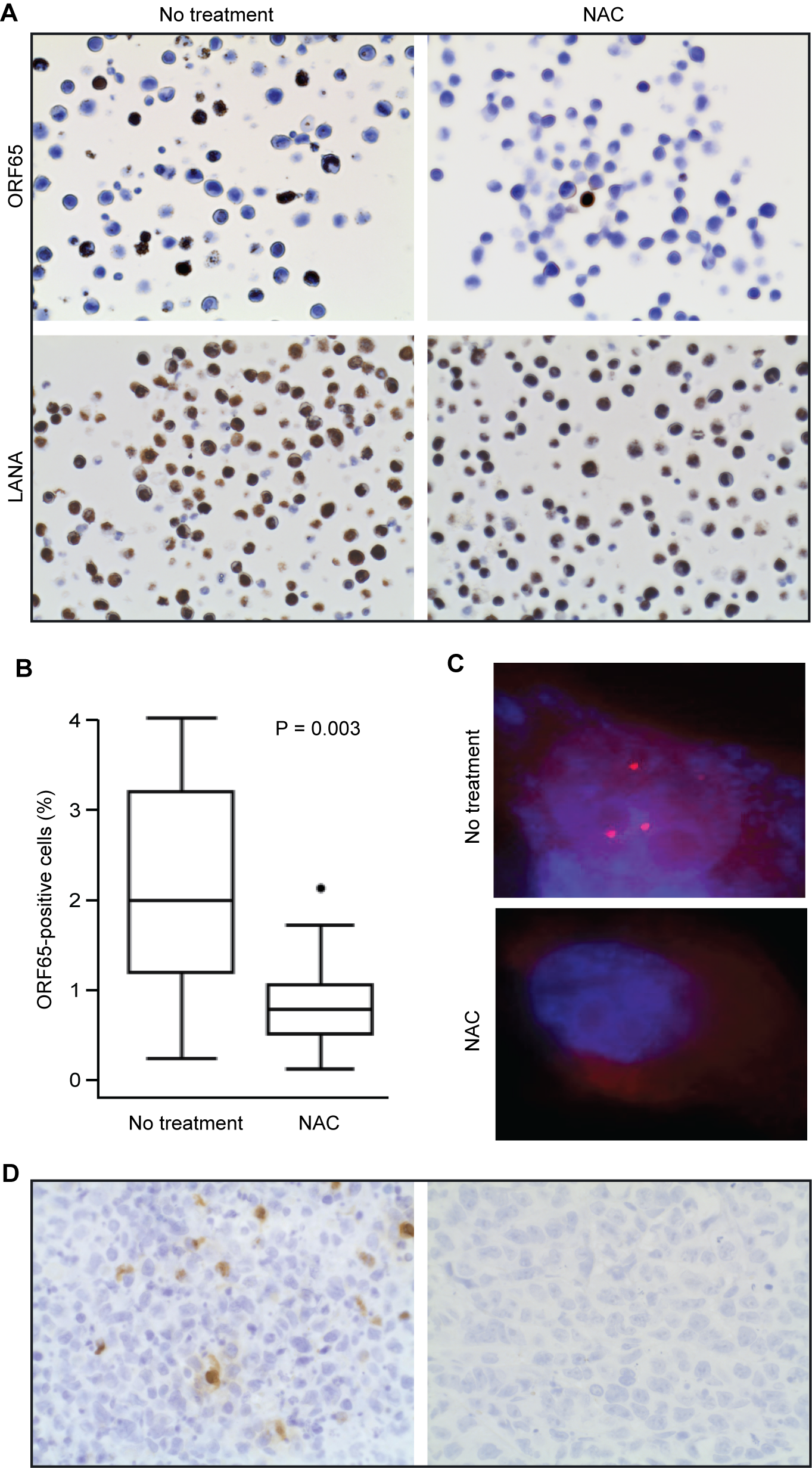

Supplement: Figure S2 — NAC treatment inhibits KSHV lytic replication in a mouse PEL model. (A) Representative immunohistochemistry images of ORF65 and LANA staining in lymphoma cells from untreated control and NAC-treated mice. (B) Percentages of ORF65-positive cells in lymphomas from untreated control and NAC-treated mice. (C) Detection of KSHV particles by ORF65 staining in endothelial cells infected with supernatants of lymphomas from untreated control and NAC-treated mice. Immunofluorescence staining was performed at 4 hpi. (D) Representative immunohistochemistry images of ORF65 staining in solid tumors from untreated control and NAC-treated mice. (TIF) [file ppat.1002054.s002.tif]
